# Combined Epicardial and Endocardial Sinus Node Modification Using the Orion™ Mini-basket Mapping Catheter

**DOI:** 10.19102/icrm.2021.120305

**Published:** 2021-03-15

**Authors:** Ziad F. Issa

**Affiliations:** ^1^Prairie Heart Institute, Springfield, IL, USA

**Keywords:** Electroanatomical mapping, epicardial ablation, inappropriate sinus tachycardia, phrenic nerve injury, sinus node modification

## Abstract

In patients with inappropriate sinus tachycardia, conservative medical management targeting the relief of symptoms is the first line of therapy. Sinus node modification can offer a potential benefit in selected patients with severe, refractory inappropriate sinus tachycardia. Extensive endocardial radiofrequency (RF) ablation of the superior aspect of the sinus node complex is typically required but is often limited by the epicardial location of the sinus node and the proximity of the phrenic nerve. More recently, surgical and catheter-based epicardial approaches to the sinus node have been used to facilitate more direct access to the sinus node and mechanical displacement of the phrenic nerve from ablation target sites. In this case report, we describe a combined epicardial–endocardial sinus node modification procedure in a patient with refractory inappropriate sinus tachycardia and previous unsuccessful endocardial ablation. The Orion™ mini-basket catheter (Boston Scientific, Natick, MA, USA) was used both for mapping the sinus node and for mechanically displacing the phrenic nerve from ablation target sites, which facilitated successful ablation.

## Case presentation

A 32-year-old man with a history of hypertension presented with chronic symptoms of palpitations, dizziness, fatigue, and dyspnea during mild activity, which had been persistent for two years. Resting sinus rates during multiple clinic visits were consistently above 140 bpm. A 24-hour Holter monitor demonstrated sinus tachycardia with an average rate of 106 bpm. Extensive evaluation failed to uncover a secondary etiology of sinus tachycardia and the patient was diagnosed with inappropriate sinus tachycardia (IST). Lifestyle changes, volume-expansion measures, and medical therapy offered little to no benefit.

Due to refractory, debilitating symptoms, the patient underwent sinus node modification. Extensive endocardial RF ablation was performed using an irrigated-tip catheter but failed to affect the sinus rate. On multiple occasions, RF ablation was prohibited due to the close proximity of the phrenic nerve (PN), as identified by PN capture during high-output pacing at ablation target sites.

A few months later, the patient underwent combined epicardial–endocardial ablation under general anesthesia. Paralytic agents were avoided to allow monitoring of PN function. A multipolar catheter was placed along the crista terminalis in the right atrium (RA) via the femoral vein. The pericardial space was then accessed percutaneously via the subxiphoid region (using the anterior approach).^1^ Two steerable sheaths (Agilis; Abbott, Chicago, IL, USA) were advanced into the pericardial space for the Orion™ (Boston Scientific, Natick, MA, USA) and ablation catheters.

**Figure 1:**
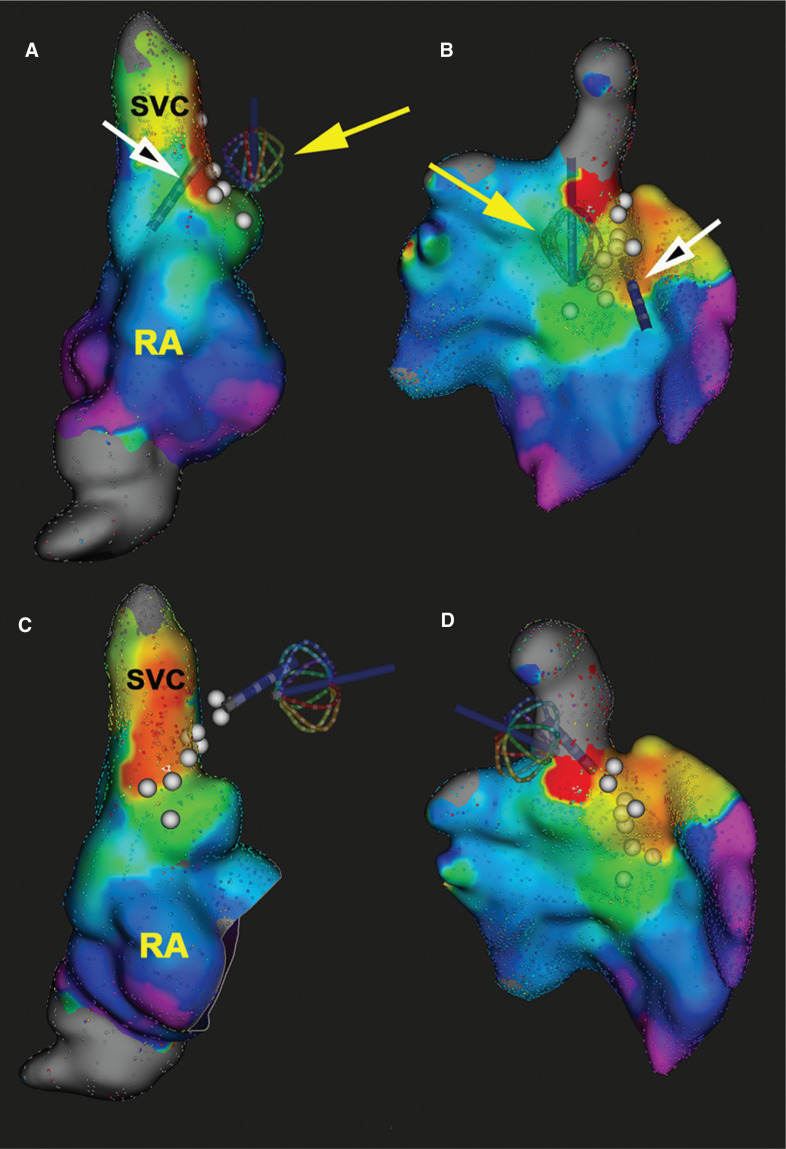
Endocardial **(A, C)** and epicardial **(B, D)** RA ultra–high-density electroanatomic activation maps created during sinus tachycardia using the Rhythmia™ electroanatomical mapping system and the Orion™ catheter. The earliest site of activation (red) was mapped to the RA–superior vena cava junction. White dots mark sites of PN capture during high-output pacing. The ablation catheter (white arrows) was positioned endocardially **(A, B)** and epicardially **(C, D)** at the site of earliest activation, where PN capture was demonstrated during high-output pacing from the tip of the ablation catheter. The Orion™ catheter (yellow arrows) was deployed in the pericardial space next to the ablation target sites to lift the PN away; repeat pacing failed to capture the PN. Ablation was then performed without injury to the PN. Note that, when the Orion™ catheter was used for epicardial mapping, the inner surface of the electroanatomical map (shown in the right lower panel) became the relevant surface as it represents the epicardial surface, while the outer surface (shown in the upper right panel) was collected by the Orion™ catheter electrodes not in direct contact with the epicardium. RA: right atrium; SVC: superior vena cava.

Initially, the Orion™ catheter was introduced via the femoral vein into the RA and mapping was performed endocardially. Activation mapping was conducted during sinus tachycardia (under maximal adrenergic stimulation with isoproterenol) using the Rhythmia™ electroanatomical mapping system (Boston Scientific, Natick, MA, USA) to identify the earliest site of activation during sinus tachycardia. Then, the Orion™ catheter was advanced into the pericardial space and epicardial activation maps were acquired. Ultra–high-density electroanatomic activation maps revealed the earliest atrial activation sites preceded the onset of the surface P-wave by 15 ms endocardially and by 24 ms epicardially **(Figure 1)**.

**Figure 2:**
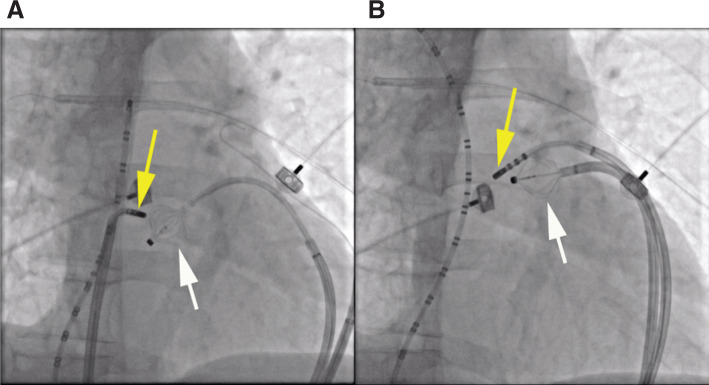
Fluoroscopy right anterior oblique views during RF ablation of the sinus node. A multipolar catheter was positioned along the crista terminalis in the right atrium, extending into the superior vena cava. The Orion™ catheter (white arrow) was positioned in the pericardial space via a deflectable sheath and deployed to mechanically lift the PN away from the ablation target sites. **A:** The ablation catheter was placed endocardially via the femoral vein. **B:** The ablation catheter was placed epicardially via a second deflectable sheath.

An irrigated-tip catheter was used for RF application, with an RF power output of 40 to 50 W, keeping the tip temperature less than 45°C. Ablation was initially performed endocardially, targeting sites with the earliest activation time, along the superior aspect of the crista terminalis. Before each application of RF energy, high-output pacing was performed from the tip of the ablation catheter to verify the absence of diaphragmatic stimulation and avoid PN injury. Sites at which pacing stimulated the diaphragm were tagged and the Orion™ catheter was subsequently advanced into the pericardial space and maneuvered to the epicardial surface opposing those sites **(Figure 1)**. Then, the basket was deployed into a semi-spherical configuration to mechanically displace the pericardium and PN away from the epicardial surface **(Figure 2)**. Pacing from the ablation catheter was then repeated to assure the absence of PN stimulation.

Endocardial RF ablation failed to significantly affect the sinus rate. Therefore, the ablation catheter was advanced into the pericardial space and RF energy was delivered epicardially. Meanwhile, the Orion™ catheter was kept in the pericardial space and maneuvered to accompany the ablation catheter to help displace the PN. Whenever a sustained change in sinus rate or P-wave axis was observed, activation mapping was repeated epicardially using the Orion™ catheter to identify new sites with the earliest atrial activation, which were then targeted by ablation. Fifty-seven RF lesions were delivered in total, with a total RF energy application time of 1,288 seconds.

At the conclusion of the ablation procedure, the patient had an accelerated junctional rhythm at 82 bpm, which later converted into sinus rhythm at 80 to 90 bpm. No intrapericardial bleeding was observed and the pericardial sheaths were removed without leaving a pericardial drain. There were no periprocedural complications. The patient was discharged on the second postoperative day. At one month of follow-up, he reported a major improvement in his symptoms and a 24-hour Holter monitor showed sinus rates ranging between 53 and 125 bpm, with an average rate of 83 bpm.

## Discussion

IST is characterized by a persistent increase in the resting sinus rate unrelated to or out of proportion with the normal physiologic demand.^2^ Currently, treatment of IST is generally palliative and conservative medical management with a multidisciplinary approach is the mainstay of therapy. For patients with debilitating symptoms in whom medical therapy is not effective or not tolerated, sinus node modification by RF ablation can be an important therapeutic option. Sinus node modification targets areas of the sinus node responsible for rapid rates while preserving some chronotropic competence and often requires complete abolition of the cranial portion of the sinus node complex.^2,3^

Various endocardial ablation techniques have been developed; however, given the predominantly epicardial location of the sinus node and its close proximity to the PN that can prohibit adequate ablation, procedural success has been limited.^4^ More recently, surgical and catheter-based epicardial approaches to the sinus node have been used to achieve more direct access to the sinus node and facilitate the use of additional techniques for mechanical displacement of the PN from ablation target sites.^5,6^

When proximity to the PN prohibits RF application at the endocardial or epicardial target sites, different strategies have been proposed to displace the PN and allow safe ablation.^1^ One technique involves the interpositioning of a large peripheral vascular angioplasty balloon (or esophageal balloon) between the heart and pericardium. An alternative technique is to inject a combination of saline and air into the pericardium to achieve a “controlled hydropneumo-pericardium” to increase the distance between the PN and the ablation target area.^1,7–9^

The Rhythmia™ three-dimensional electroanatomical mapping platform paired with the Orion™ catheter is capable of generating ultra–high-density electroanatomic maps. The Orion™ catheter is an 8.5-French bidirectional deflectable catheter with a mini-basket electrode array containing eight splines, with each spline containing eight small electrodes. The basket can be deployed into a spherical configuration through the mechanical flexion of the splines to varying diameters (i.e., minimum: 3 mm vs. nominal: 18 mm vs. maximum: 22 mm, when measured at its equator).

In our patient, the Orion™ catheter facilitated the quick acquisition of endocardial and epicardial activation maps as well as mechanical displacement of the PN away from ablation target sites. During most of the procedure, the Orion™ catheter was kept in the pericardial space and was moved in tandem with the ablation catheter and deployed whenever ablation was attempted endocardially or epicardially. Additionally, the Orion™ catheter was in a position to quickly acquire new epicardial activation maps when sinus rates changed to guide subsequent ablation. The steerability of the Orion™ catheter is superior to that of a peripheral angioplasty balloon (even when used with a steerable sheath) and, hence, the Orion™ catheter is easier to maneuver to the desired epicardial location. Furthermore, unlike the angioplasty balloon, the Orion™ catheter can be visualized on the electroanatomical map, facilitating its positioning at the desired sites identified during mapping without the need for fluoroscopy.

On the other hand, the use of “controlled hydropneumopericardium” could potentially be limited by the poor hemodynamic tolerance in this case, given the fact that the patient was under general anesthesia and receiving a high dose of intravenous isoproterenol. In addition, air is a poor conductor of electricity and its presence in the pericardial space can increase the defibrillation threshold.^7,9,10^
